# Consistent effects of non-invasive vagus nerve stimulation (nVNS) for the acute treatment of migraine: additional findings from the randomized, sham-controlled, double-blind PRESTO trial

**DOI:** 10.1186/s10194-018-0929-0

**Published:** 2018-11-01

**Authors:** Paolo Martelletti, Piero Barbanti, Licia Grazzi, Giulia Pierangeli, Innocenzo Rainero, Pierangelo Geppetti, Anna Ambrosini, Paola Sarchielli, Cristina Tassorelli, Eric Liebler, Marina de Tommaso, on Behalf of the PRESTO Study Group, Cristina Tassorelli, Vito Bitetto, Roberto De Icco, Daniele Martinelli, Grazia Sances, Monica Bianchi, Licia Grazzi, Anna Maria Padovan, Marina de Tommaso, Katia Ricci, Eleonora Vecchio, Pietro Cortelli, Sabina Cevoli, Giulia Pierangeli, Rossana Terlizzi, Paolo Martelletti, Andrea Negro, Gabriella Addolorata Chiariello, Innocenzo Rainero, Paola De Martino, Annalisa Gai, Flora Govone, Federica Masuzzo, Elisa Rubino, Maria Claudia Torrieri, Alessandro Vacca, Pierangelo Geppetti, Alberto Chiarugi, Francesco De Cesaris, Simone Li Puma, Chiara Lupi, Ilaria Marone, Anna Ambrosini, Armando Perrotta, Paola Sarchielli, Laura Bernetti, Ilenia Corbelli, Michele Romoli, Simone Simoni, Angela Verzina, Piero Barbanti, Cinzia Aurilia, Gabriella Egeo, Luisa Fofi, Eric Liebler, Annelie Andersson, Lia Spitzer, Juana Marin, Candace McClure, Lisa Thackeray, Maria Giovanna Baldi, Daniela Di Maro

**Affiliations:** 1 Department of Clinical and Molecular Medicine, Sapienza University, Rome, Italy; 2 Headache and Pain Unit, Istituto di Ricovero e Cura a Carattere Scientifico (IRCCS) San Raffaele Pisana, Rome, Italy; 3 Neuroalgology Unit, Carlo Besta Neurological Institute and Foundation, Milan, Italy; 4 IRCCS Istituto delle Scienze Neurologiche di Bologna, Bologna, Italy; 5 Department of Neuroscience, University of Turin, Turin, Italy; 6 Headache Centre, University Hospital of Careggi, Florence, Italy; 7 IRCCS Neuromed, Pozzilli, IS Italy; 8 Neurologic Clinic, Santa Maria della Misericordia Hospital, Perugia, Italy; 9 Headache Science Centre, IRCCS C. Mondino Foundation, Pavia, Italy; 10 Department of Brain and Behavioral Sciences, University of Pavia, Pavia, Italy; 11 electroCore, Inc., Basking Ridge, NJ USA; 12 Neurophysiology and Pain Unit, University of Bari Aldo Moro, Bari, Italy

**Keywords:** Neuromodulation, Vagus nerve stimulation, Migraine, Pain intensity, Double-blind, Open-label

## Abstract

**Background:**

Non-invasive vagus nerve stimulation (nVNS) has been shown to be practical, safe, and well tolerated for treating primary headache disorders. The recent multicenter, randomized, double-blind, sham-controlled PRESTO trial provided Class I evidence that for patients with episodic migraine, nVNS significantly increases the probability of having mild pain or being pain-free 2 h post stimulation. We report additional pre-defined secondary and other end points from PRESTO that demonstrate the consistency and durability of nVNS efficacy across a broad range of outcomes.

**Methods:**

After a 4-week observation period, 248 patients with episodic migraine with/without aura were randomly assigned to acute treatment of migraine attacks with nVNS (*n* = 122) or a sham device (*n* = 126) during a double-blind period lasting 4 weeks (or until the patient had treated 5 attacks). All patients received nVNS therapy during the subsequent 4-week/5-attack open-label period.

**Results:**

The intent-to-treat population consisted of 243 patients. The nVNS group (*n* = 120) had a significantly greater percentage of attacks treated during the double-blind period that were pain-free at 60 (*P* = 0.005) and 120 min (*P* = 0.026) than the sham group (*n* = 123) did. Similar results were seen for attacks with pain relief at 60 (*P* = 0.025) and 120 min (*P* = 0.018). For the first attack and all attacks, the nVNS group had significantly greater decreases (vs sham) in pain score from baseline to 60 min (*P =* 0.029); the decrease was also significantly greater for nVNS at 120 min for the first attack (*P* = 0.011). Results during the open-label period were consistent with those of the nVNS group during the double-blind period. The incidence of adverse events (AEs) and adverse device effects was low across all study periods, and no serious AEs occurred.

**Conclusions:**

These results further demonstrate that nVNS is an effective and reliable acute treatment for multiple migraine attacks, which can be used safely while preserving the patient’s option to use traditional acute medications as rescue therapy, possibly decreasing the risk of medication overuse. Together with its practicality and optimal tolerability profile, these findings suggest nVNS has value as a front-line option for acute treatment of migraine.

**Trial registration:**

ClinicalTrials.gov identifier: NCT02686034.

## Background

Standard pharmacologic agents for the acute treatment of migraine can be limited by side effects, inconsistent efficacy, contraindications, risk of drug interactions, and their potential contribution to migraine chronification and medication overuse headache [1–5]. Opioids should be discouraged for the acute treatment of migraine due to significant safety concerns and lack of documented efficacy but remain frequently used in the emergency department setting, which significantly increases healthcare costs [6–9]. Practical alternatives are needed to address this healthcare challenge. Non-invasive neuromodulation therapies could represent a novel option for these patients [10, 11].

Non-invasive vagus nerve stimulation (nVNS; gammaCore®; electroCore, Inc., Basking Ridge, NJ, USA) demonstrated efficacy in studies of acute migraine treatment and has a strong safety and tolerability profile [12–15]. The multicenter, randomized, double-blind, sham-controlled PRESTO trial provided Class I evidence that for patients with an episodic migraine, acute treatment of migraine attacks with nVNS significantly increases the probability of having mild pain or being pain-free 2 h post stimulation [11]. The study also clearly demonstrated the practicality, safety, and tolerability of nVNS. Here, we report additional pre-defined secondary and other end points from the PRESTO study to illustrate the consistency and durability of nVNS effects across a broad range of outcomes.

## Methods

### Study design

**Fig. 1:**
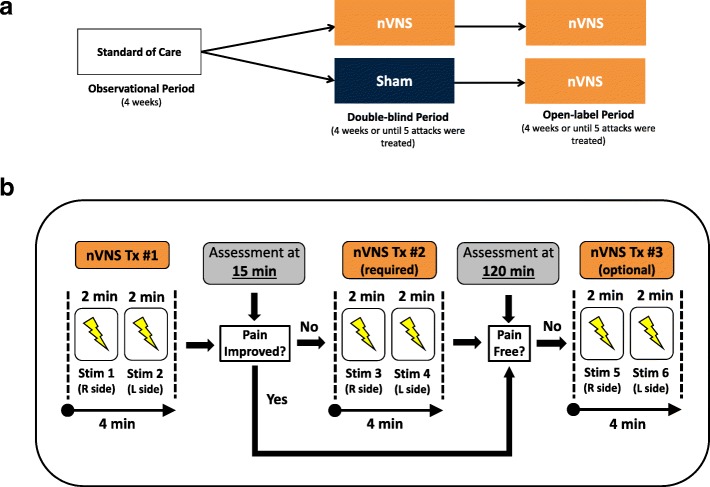
PRESTO study design (**a**) and treatment protocol (**b**). Abbreviations: L, left; nVNS, non-invasive vagus nerve stimulation; R, right; Stim, stimulation; Tx, treatment

Complete details of the methodology of the multicenter, randomized, double-blind, sham-controlled PRESTO trial have been reported previously [11]. The study was conducted across 10 Italian sites from January 11, 2016, through March 31, 2017, and consisted of an observational period, double-blind period, and open-label period (Fig. 1a). During the observational period, patients treated their migraine attacks with standard medications according to their individual prescriptions. Patients subsequently treated up to 5 migraine attacks with nVNS or sham stimulation during the double-blind period and up to 5 additional attacks with nVNS during the open-label period; only 1 attack could be treated in a 48-h period.

### Patients

Study patients were 18 to 75 years of age, had a previous diagnosis of migraine with or without aura according to the International Classification of Headache Disorders, 3rd edition (beta version) criteria [16], were < 50 years of age at migraine onset, and had 3 to 8 migraine attacks per month with < 15 headache days per month during the last 6 months. Patients who were receiving preventive migraine medications at baseline (or other preventive medications determined to potentially interfere with the study) were required to have maintained a stable dose and frequency of these medications during the 2 months before enrollment and throughout the study; initiation of new preventive medications was not permitted during this period.

### Interventions and study procedures

Patients were randomly assigned (1:1) to receive nVNS or sham (variable block design [4, 6], stratified by site). Full details of study randomization and blinding, as well as information about the active and sham devices, have been described previously [11].

Within 20 min of migraine pain onset, patients self-administered bilateral 120-s stimulations (ie, 1 stimulation each to the right and left sides of the neck) (Fig. 1b) and recorded post-treatment assessments in their study diaries 15, 30, 60, and 120 min and 24 and 48 h after completion of the initial bilateral stimulations. Patients were instructed to repeat the bilateral stimulations if pain had not improved at the 15-min assessment, and those who were not pain-free at the 120-min assessment had the option of administering an additional set of bilateral stimulations. Patients were asked to wait 120 min from the first set of stimulations before using acute rescue medication.

### End points

Here, we present clinically relevant secondary and other end points not included in the original PRESTO publication to provide a more comprehensive depiction of the data set. *Pain-free* was defined as a score of 0 on the 4-point headache pain scale (with 0 indicating no pain and 3 indicating severe pain), and *pain relief* was defined as a score of 0 or 1 (both without use of rescue medication before 120 min) in a subject with pain of at least moderate severity at baseline. Attacks with mild pain (ie, a pain score of 1) at both baseline and the subsequent time point of interest were considered treatment failures. These definitions were used in assessment of the following end points:

- Percentages of all treated attacks that achieved pain freedom and pain relief at 30, 60, and 120 min for the double-blind period and at 120 min for the open-label period;
- Mean change in pain score from baseline to 30, 60, and 120 min for the first attack and for all attacks in the double-blind and open-label periods;
- Number of acute medications used per migraine attack during the observational, double-blind, and open-label periods;
- S*ustained treatment response* (defined as pain-free or pain relief [without use of rescue medication] at both 2 h and 24 h or at 2, 24, and 48 h) rates for the first attack and all attacks for the double-blind and open-label periods;
- Incidence of adverse events (AEs) and adverse device effects (ADEs).

### Statistical methods

All efficacy end points were evaluated using the *intent-to-treat (ITT) population*, defined as all randomly assigned patients who treated at least 1 migraine attack in the double-blind period. Descriptive statistics were used to summarize continuous variables (means and 95% confidence intervals [CIs]) and categorical variables (frequency counts, percentages, and 95% CIs). Generalized linear mixed effects regression models were used to estimate the proportion of all attacks that were pain-free or had pain relief for the nVNS and sham groups, allowing for both subject-specific and population-averaged inferences in non-normally distributed data; *P* values were from resulting F tests. Mean change from baseline pain score was compared between treatment groups via 2-sample *t* tests for the first attack and via linear mixed effects regression models. Poisson regression was used to compare medication use per attack between treatment groups. For sustained treatment response (pain-free and pain relief), the nVNS and sham groups were compared via the chi-square test or Fisher exact test, as appropriate, for the first attack and via linear mixed effects regression models for all attacks. All data were analyzed using SAS®9.4 (SAS Institute Inc., Cary, NC, USA). Two-sided *P* values < 0.05 were considered statistically significant.

## Results

### Patients

Complete descriptions of patient disposition, demographics, and baseline characteristics in PRESTO are included in the original study publication [11]. The ITT population consisted of 243 patients (nVNS, *n* = 120; sham, *n* = 123). Two hundred thirty-nine patients entered the open-label period (nVNS, *n* = 117; sham, *n* = 122); among these, 238 patients (> 99%) completed this period (1 patient was lost to follow-up), with 220 (92%) treating at least 1 attack during the period. One patient who treated at least 1 attack in and completed the open-label period was not part of the ITT population. Table 1 summarizes key demographics and other key patient characteristics.

**Table 1:** Demographics and other key patient characteristics

Characteristic	nVNS (*n* = 120)	Sham (*n* = 123)	Total (*N* = 243)
Age, mean (SD), y	38.8 (11.0)	39.6 (11.8)	39.2 (11.4)
Female sex, No. (%)	95 (79.2)	91 (74.0)	186 (76.5)
Diagnosis, No. (%)			
Migraine with aura	8 (6.7)	9 (7.3)	17 (7.0)
Migraine without aura	112 (93.3)	114 (92.7)	226 (93.0)
Current preventive medication use, No. (%)	42 (35.0)	35 (28.5)	77 (31.7)
No. of acute medication days per mo,^a^ mean (SD)	5.6 (1.7)	5.3 (1.7)	5.5 (1.7)
Attack severity at onset for all treated attacks in DB period, No. (%)	*n = 359* ^b^	*n = 329* ^b^	NA
Mild	113 (31.5)	105 (31.9)	
Moderate	156 (43.5)	166 (50.5)	
Severe	90 (25.1)	58 (17.6)	
Attack severity at onset for first treated attack in DB period, No. (%)	*n = 119* ^b^	*n = 119* ^b^	NA
Mild	40 (33.6)	46 (38.7)	
Moderate	51 (42.9)	55 (46.2)	
Severe	28 (23.5)	18 (15.1)	

Tassorelli C, Grazzi L, de Tommaso M, et al. Non-invasive vagus nerve stimulation as acute therapy for migraine: the randomized PRESTO study. Neurology. 2018;91(4):e364−e373

Abbreviations: *DB* Double-blind, *NA* Not applicable, *nVNS* Non-invasive vagus nerve stimulation, *SD* Standard deviation

^a^No. of days the patient typically takes acute migraine medication per month. ^b^ Patients with no reported baseline severity were excluded from this analysis

### Efficacy: double-blind period

**Fig. 2:**
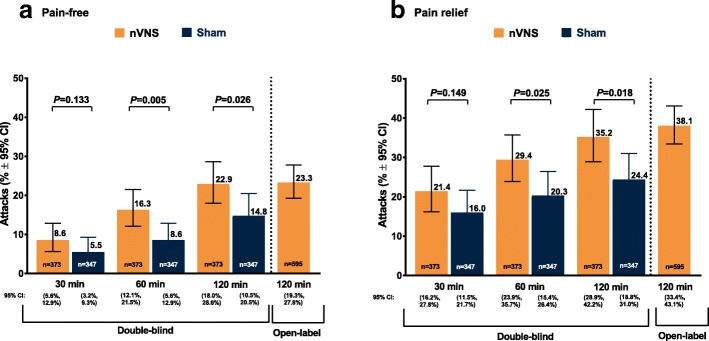
Percentage of all treated attacks that were pain-free (**a**) or had pain relief (**b**) during the double-blind and open-label periods (ITT population, *N* = 243). Generalized linear mixed effects regression models were used to estimate the proportion of successful responses, allowing for both subject-specific and population-averaged inferences in non-normally distributed data. *P* values are from resulting F tests. Models were adjusted for subject’s pain score at baseline, use of preventive therapies, and indicator or presence of aura. Abbreviations: CI, confidence interval; ITT, intent-to-treat; nVNS, non-invasive vagus nerve stimulation

In the nVNS group, the percentage of all attacks that were pain-free at 60 min (16.3%) and 120 min (22.9%) was significantly greater than in the sham group (8.6% and 14.8%, respectively; *P* < 0.05 for both time points) (Fig. 2a). Similar significant results were seen for the percentage of attacks with pain relief (Fig. 2b), which was 29.4% for the nVNS group and 20.3% for the sham group at 60 min (*P* = 0.025) and 35.2% for the nVNS group and 25.4% for the sham group at 120 min (*P* = 0.018).

**Fig. 3:**
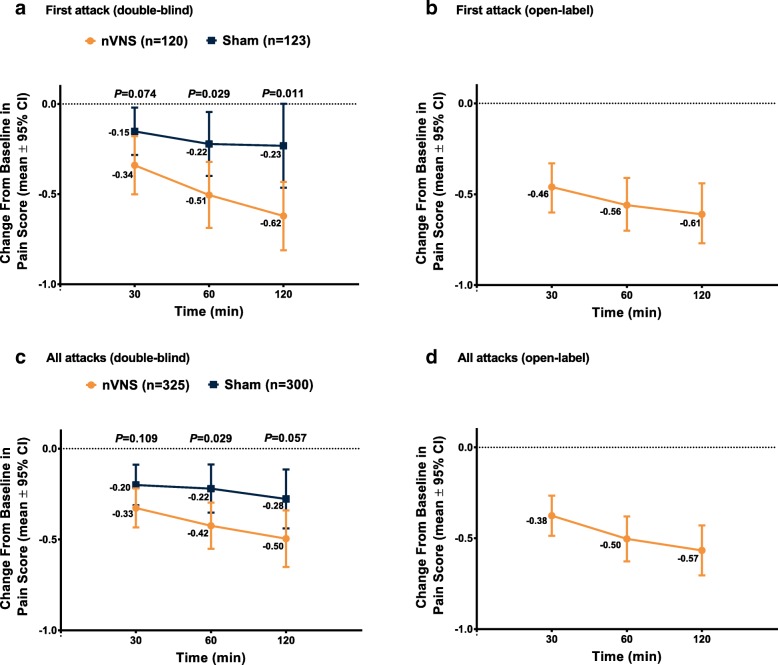
Mean change in pain score from baseline for first attack during the double-blind (**a**) and open-label (**b**) periods and for all attacks during the double-blind (**c**) and open-label Periods (**d**). *P* values for first attack are from 2-sample *t* tests. For all attacks, linear mixed effects regression models were used to estimate the change in pain score between baseline and 30, 60, and 120 min, allowing for both subject-specific and population-averaged inferences. Abbreviations: CI, confidence interval; nVNS, non-invasive vagus nerve stimulation

For the first attack (Fig. 3a), the nVNS group had significantly greater decreases (vs the sham group) in mean pain score from baseline at 60 min (nVNS, 0.51; sham, 0.22; *P* = 0.029) and 120 min (nVNS, 0.62; sham, 0.23; *P* = 0.011). For all attacks (Fig. 3c), the mean decrease from baseline in pain score was significantly greater in the nVNS group (0.42) than in the sham group (0.22) at 60 min (*P* = 0.029) but not at 120 min (nVNS, 0.50; sham, 0.28; *P* = 0.057).

During the observational (run-in) period, study patients used a mean of 0.86 acute medications per attack. Acute medication use decreased during the double-blind period to 0.45 medications per attack in the nVNS group and 0.55 medications per attack in the sham group (*P* = 0.055).

Sustained pain-free and pain relief response rates were high in both the nVNS and sham groups at 24 h (≥75%) and 48 h (≥58%) for both the first attack and all attacks (Table 2).

**Table 2:** Sustained response at 24 and 48 h post-treatment

	Double-blind Period			Open-label Period
	nVNS	Sham	*P* Value	nVNS
	% (n/N) (95% CI)	% (n/N) (95% CI)		% (n/N) (95% CI)
All Attacks				
Pain-free				
24 h	79.0 (71/93) (67.9, 87.1)	83.7 (54/66) (69.9, 91.9)	0.532^a^	80.8 (118/146) (73.5, 86.6)
48 h	65.7 (59/93) (53.3, 76.3)	71.2 (46/66) (56.5, 82.5)	0.537^a^	70.3 (102/146) (61.6, 77.7)
Pain relief				
24 h	80.9 (109/138) (72.3, 87.3)	80.3 (78/99) (69.4, 87.9)	0.916^a^	80.5 (185/230) (74.5, 85.3)
48 h	74.1 (99/138) (64.0, 82.1)	72.1 (70/99) (60.7, 81.3)	0.775^a^	71.9 (165/230) (65.2, 77.7)
First Attack				
Pain-free				
24 h	75.0 (27/36) (57.8, 87.9)	84.6 (22/26) (65.1, 95.6)	0.359^b^	75.4 (46/61) (62.7, 85.5)
48 h	58.3 (21/36) (40.8, 74.5)	69.2 (18/26) (48.2, 85.7)	0.381^b^	65.6 (40/61) (52.3, 77.3)
Pain relief				
24 h	77.3 (58/75) (66.2, 86.2)	79.3 (46/58) (66.7, 88.8)	0.784^b^	78.8 (108/137) (71.0, 85.3)
48 h	69.3 (52/75) (57.6, 79.5)	71.0 (41/58) (57.3, 81.9)	0.866^b^	70.1 (96/137) (61.7, 77.6)

Abbreviations: *CI* Confidence interval, *nVNS* Non-invasive vagus nerve stimulation

^a^Generalized linear mixed effects regression models were used to estimate the proportion of successful responses, allowing for both subject-specific and population-averaged inferences in non-normally distributed data. ^b^From chi-square test or Fisher exact test, as appropriate

### Efficacy: open-label period

The percentages of all treated attacks that were pain-free (23.3%) or had pain relief (38.1%) at 120 min during the open-label period were similar to those of the nVNS group during the double-blind period (Fig. 2). Mean changes from baseline in pain score during the open-label period for the first attack (30 min, −or t; 60 min, − 0.56; 120 min, − 0.61) and for all treated attacks (30 min, − 0.38; 60 min, − 0.50; 120 min, − 0.57) were similar to those seen in the nVNS group during the double-blind period (Fig. 3). A mean of 0.46 acute medications per attack were used during the open-label period, which was similar to that of the nVNS group during the double-blind period (0.45 acute medications per attack) and was decreased from the observation period by 0.40 acute medications per attack. Sustained treatment response during the open-label period was generally similar to or greater than that seen in the nVNS group during the double-blind period (Table 2).

### Safety

As previously reported [11], the incidence of AEs and ADEs was low across all study periods, and no serious AEs occurred. The only ADE reported by > 1 patient during the open-label period was vertigo, which was reported by 2 patients (1%).

## Discussion

These additional results from the PRESTO study further demonstrate that nVNS is superior to sham across a broad range of relevant end points. In the nVNS group, significantly greater percentages of all attacks were pain-free or had pain relief at 60 and 120 min than in the sham group. The nVNS group also had significantly greater decreases from baseline in mean pain score for the first attack (60 and 120 min) and for all attacks (60 min). Among nVNS-treated attacks that were pain-free at 120 min, > 75% had a sustained response at 24 h. Ninety-eight percent of patients in the ITT population completed the open-label period, suggesting that the benefit from nVNS was maintained and that nVNS is a durable acute therapy. The results for the total population during the open-label period were generally similar to those seen for the nVNS group during the double-blind period. Throughout the study, the incidence of AEs and ADEs was low, and no serious AEs were reported.

The findings from PRESTO are consistent with those from other clinical studies of acute nVNS use in migraine [12, 13]. They are supported by several potential mechanisms of action for the acute benefits of vagus nerve stimulation, including inhibition of central excitability through suppression of glutamate release, suppression of acute nociceptive activation of trigeminocervical neurons, and curbing expression of proteins associated with central sensitization of trigeminal neurons [17–19].

The high rates of sustained 24-h pain-free response to nVNS seen in PRESTO (> 75%) stand in contrast to the lower rates reported for oral triptans (10%–30%) and single-pulse transcranial magnetic stimulation (29%) [10, 20]. The protocol of the single-pulse transcranial magnetic stimulation study called for a study population restricted to patients with aura and a treatment time that was independent from the onset of pain (ie, within 1 h after aura onset), making comparison with the nVNS findings challenging [10]. The majority of patients enrolled in the PRESTO study had migraine without aura, and nVNS was delivered promptly after the onset of migraine pain (ie, within 20 min) [11]. In the majority of the triptan clinical trials, treatment was not initiated until migraine pain reached a moderate/severe level, partially due to the desire to avoid unnecessary adverse effects [20, 21]. Consistent with findings from PRESTO [22], rates of sustained response appear to be higher with the use of triptans during the early stages of migraine (34%–53%) than during the later stages (19%–31%) [21, 23]. These observations suggest that there are benefits to treating early in the course of migraine attacks—in a sense, intervening before the migraine process is fully activated. High rates of sustained pain-free response in both the nVNS and sham groups in PRESTO suggest that intervention early in the course of migraine might confer benefits, irrespective of the treatment.

Together with findings from multiple previous studies [12–15, 24–26], these results from PRESTO further highlight the clinical utility, practicality, and flexibility of nVNS. Across the double-blind and open-label periods, nVNS was used to treat > 900 migraine attacks, with data collected at multiple time points for each attack, demonstrating its consistent efficacy, safety, and tolerability as acute treatment for these attacks. nVNS can be used as monotherapy or in conjunction with other treatments without risk of pharmacologic interactions, offering a clinical versatility that other acute migraine treatments lack. These advantages, along with its convenience and ease of use, make nVNS an appealing and pragmatic option for early, adjunctive, and/or frequent use in the acute treatment of migraine. nVNS could also help minimize the risk of medication overuse associated with traditional acute treatments and reduce the frequency of opioid use for the acute treatment of migraine in the emergency department setting.

This study has a number of limitations. The selection of an appropriate sham device in neuromodulation studies is challenging. In accordance with previous recommendations to ensure maintenance of the study blind [27], the sham device used in PRESTO produced an active signal that could be perceived by the user but was not designed to stimulate the vagus nerve; recent data suggest that the strength of the sham device’s signal may have inadvertently activated the vagus nerve and could have inflated the responses to sham treatment across all end points [28]. This phenomenon, which merits further investigation, may have been related to a psychobiological placebo effect but more likely resulted from the unanticipated physiologically active signal that may have decreased the difference in therapeutic gain seen between the nVNS and sham groups [11].

During both the double-blind and open-label periods, the mean number of acute medications used per migraine attack was substantially lower than that seen during the observational period. Such a decrease in medication use could be interpreted as evidence of treatment efficacy; however, these results must be interpreted with caution, as patients were encouraged to refrain from using acute medications for 120 min after stimulation with the study device. This study limitation most likely contributed to decreases in acute medication use in both the nVNS and sham groups during the double-blind period and may partially explain the lack of significance between treatment groups for this end point.

## Conclusions

These results from clinically relevant secondary and other end points of the PRESTO study demonstrate the efficacy and reliability of nVNS for the acute treatment of migraine. nVNS provided dependable efficacy for the successful treatment of multiple attacks and can be used safely while preserving a patient’s option to use additional acute medications as rescue therapy, thus potentially decreasing the risk of medication overuse. Together, these findings highlight the flexibility and practicality of nVNS as a front-line option for acute migraine attacks.

## Abbreviations

ADE: Adverse device effect
AE: Adverse event
CI: Confidence interval
DB: Double-blind
ITT: Intent to treat
L: Left
NA: Not applicable
nVNS: Non-invasive vagus nerve stimulation
R: Right
SD: Standard deviation
Stim: Stimulation
Tx: Treatment
