# Correction to: Consistent effects of non-invasive vagus nerve stimulation (nVNS) for the acute treatment of migraine: additional findings from the randomized, sham-controlled, double-blind PRESTO trial

**DOI:** 10.1186/s10194-018-0949-9

**Published:** 2018-12-18

**Authors:** Paolo Martelletti, Piero Barbanti, Licia Grazzi, Giulia Pierangeli, Innocenzo Rainero, Pierangelo Geppetti, Anna Ambrosini, Paola Sarchielli, Cristina Tassorelli, Eric Liebler, Marina de Tommaso, on Behalf of the PRESTO Study Group

**Affiliations:** 1 Department of Clinical and Molecular Medicine, Sapienza University, Rome, Italy; 2 Headache and Pain Unit, Istituto di Ricovero e Cura a Carattere Scientifico (IRCCS) San Raffaele Pisana, Rome, Italy; 3 Neuroalgology Unit, Carlo Besta Neurological Institute and Foundation, Milan, Italy; 4 IRCCS Istituto delle Scienze Neurologiche di Bologna, Bologna, Italy; 5 Department of Neuroscience, University of Turin, Turin, Italy; 6 Headache Centre, University Hospital of Careggi, Florence, Italy; 7 IRCCS Neuromed, Pozzilli, IS Italy; 8 Neurologic Clinic, Santa Maria della Misericordia Hospital, Perugia, Italy; 9 Headache Science Centre, IRCCS C. Mondino Foundation, Pavia, Italy; 10 Department of Brain and Behavioral Sciences, University of Pavia, Pavia, Italy; 11 electroCore, Inc., Basking Ridge, NJ USA; 12 Neurophysiology and Pain Unit, University of Bari Aldo Moro, Bari, Italy

## Correction

Following publication of the original article [1], the authors notified us that the citation within the Table 1 legend was not presented as initially requested. Also, the word “efficacy” was missed from the background section.

The original publication has been corrected. The incorrect and correct table citations as well as background information are presented below.

- Originally published citation:

© 2018 Tassorelli C, Grazzi L, de Tommaso M, Pierangeli G, Martelletti P, Rainero I, Dorlas S, Geppetti P, Ambrosini A, Sarchielli P, Liebler E, Barbanti P, PRESTO Study Group (2018) Non-invasive vagus nerve stimulation as acute therapy for migraine: the randomized PRESTO study [published online June 15, 2018]. Neurology: 10.1212/WNL.0000000000005857. www.neurology.org. Adapted with permission

Abbreviations: *DB* Double-blind, *NA* Not applicable, *nVNS* Non-invasive vagus nerve stimulation, *SD* Standard deviation

^a^No. of days the patient typically takes acute migraine medication per month. ^b^Patients with no reported baseline severity were excluded from this analysis

- Corrected citation:

© 2018 Tassorelli C, Grazzi L, de Tommaso M, et al. Noninvasive vagus nerve stimulation as acute therapy for migraine: the randomized PRESTO study. Neurology. 2018;91(4):e364-e373. Adapted with permission

- Original Background paragraph:

Opioids should be discouraged for the acute treatment of migraine due to significant safety concerns and lack of documented but remain frequently used in the emergency department setting, which significantly increases healthcare costs

- Corrected Background paragraph:

Opioids should be discouraged for the acute treatment of migraine due to significant safety concerns and lack of documented **efficacy** but remain frequently used in the emergency department setting, which significantly increases healthcare costs
